# Chemoprophylaxis in Contacts of Patients with Cholera: Systematic Review and Meta-Analysis

**DOI:** 10.1371/journal.pone.0027060

**Published:** 2011-11-15

**Authors:** Ludovic Reveiz, Evelina Chapman, Pilar Ramon-Pardo, Tracey Perez Koehlmoos, Luis Gabriel Cuervo, Sylvain Aldighieri, Amy Chambliss

**Affiliations:** 1 Public Policies and Research for Health - Health Systems based on Primary Health Care, Pan American Health Organization/World Health Organization, Washington, District of Columbia, United States of America; 2 International Health Regulations, Alert and Response and Epidemic Diseases - Pan American Health Organization/World Health Organization, Washington, District of Columbia, United States of America; 3 Health & Family Planning Systems Programme, International Centre for Diarrheal Disease Research, Dhaka, Bangladesh; 4 Epidemic Alert and Response Team, PAHO - Communicable Diseases Unit, Pan American Health Organization/World Health Organization, Washington, District of Columbia, United States of America; 5 Department of International Health, Georgetown University School of Nursing and Health Studies, Washington, District of Columbia, United States of America; Menzies School of Health Research, Australia

## Abstract

**Introduction:**

There is a pressing need for effective measures to prevent the spread of cholera. Our systematic review assesses the effects of chemoprophylaxis in preventing cholera among exposed contacts.

**Methods and Findings:**

We considered published and unpublished reports of studies up to July 2011. For this we searched: PubMed (1966 to July, 2011), Embase (1980 to July 2011), Cochrane Central Register of Controlled Trials (6; 2011), LILACS (1982 to July, 2011), the International Clinical Trials Registry Platform (July 2011) and references of identified publications. We included controlled clinical trials (randomized and non-randomized) in which chemoprophylaxis was used to prevent cholera among patient contacts. The main outcome measures were hospitalization and laboratory diagnosis of cholera in contacts for cholera patients. We assessed the risk of bias. We identified 2638 references and these included 2 randomized trials and 5 controlled trials that added up to a total of 4,154 participants. The risk of bias scored high for most trials. The combined results from two trials found that chemoprophylaxis reduced hospitalization of contacts during the follow-up period by 8–12 days (2826 participants; RR 0.54 95% CI 0.40–0.74;I^2^ 0%). A meta-analysis of five trials found a significant reduction in disease among contacts with at least one positive sample who received chemoprophylaxis during the overall follow-up (range 4–15 days) (1,414 participants; RR 0.35 95% CI 0.18–0.66;I^2^ 74%). A significant reduction in the number of positive samples was also found with chemoprophylaxis (3 CCT; 6,918 samples; RR 0.39 95% CI 0.29–0.51;I^2^ 0%).

**Conclusion:**

Our findings suggest that chemoprophylaxis has a protective effect among household contacts of people with cholera but the results are based on studies with a high risk of bias. Hence, there is a need for adequate reliable research that allows balancing benefits and harms by evaluating the effects of chemoprophylaxis.

## Introduction

As we advance into the 21st century, an estimated one billion people remain without adequate access to safe water and sanitation and vulnerable to cholera epidemics [1]. It is estimated that there are 3–5 million cholera cases every year, leading to 100,000–130,000 deaths, mostly in Africa and Asia, and affecting both children and adults [2], [3]. The growing number of people affected involving major cholera outbreaks are cause for concern at the World Health Organization (WHO); WHO reported a 24% an increase in reported cases for the 2004 to 2008 period compared to the 2000 to 2004 period [4]. Most affected countries report an overall cholera case-fatality rate (CFR) under 5%, but in some locations the CFR approaches 50% during outbreaks, affecting highly vulnerable groups [3].

Currently (2011) the world is facing the so called “seventh cholera pandemic” that began in Indonesia in 1961 and is caused by an El Tor biotype of Vibrio cholera serogroup O1. An outbreak has happened in Haiti and it is of particular concern because of the devastation associated to the earthquake on 12 January 2010 that dramatically increased the vulnerability to the spread of cholera [5]. The strains of Vibrio cholera found in Haiti belong to a category known as hybrids which produce the classical type of cholera toxin and are a variant of the El Tor biotype [6]. According to the Ministère de la Santé Publique et de la Population (MSPP) of Haiti, from mid October 2010 to the third week of June 2011, a total of 363,117 cholera cases were reported in the country, of which 55% (191,508) were hospitalized and 5,506 died; the overall case fatality rate was 1.5% [7].

There is a pressing need for effective measures to prevent the spread of cholera. Although there are effective and efficient preventive measures, consisting of providing adequate access to safe water and sanitation, health education and proper food hygiene, in many settings such basic measures are difficult to implement.

The Strategic Advisory Group of Experts (SAGE) on immunization recommended the use of immunization with cholera vaccines in conjunction with other prevention and control strategies, in areas where the disease is endemic [8]. Chemoprophylaxis refers to the administration of medication to prevent disease or infection. In the case of cholera, healthy individuals are given antibiotics with the aim of protecting them against the disease, limiting the spread of the disease and curtailing an epidemic. Multiple infections in the same household are common due to shared sources of contaminated water and food. WHO does not recommend chemopophylaxis arguing that “routine treatment of a community with antibiotics, or mass chemoprophylaxis, has no effect on the spread of cholera, can have adverse effects by increasing antimicrobial resistance and provides a false sense of security” [9]. In addition, chemoprophylaxis with antibiotics is also limited by access, costs, and contraindications [9], [10].

Nevertheless, large-scale selective antibiotic prophylaxis has been provided to the contacts of people with cholera during outbreaks, as part of comprehensive community interventions [10]. Some experts argue that a well-targeted antibiotic prophylaxis can reduce direct human transmission of cholera [11], but it is difficult to evaluate the role of chemoprophylaxis in limiting cholera epidemics.

We conducted this systematic review of the findings of randomized clinical trials (RCT) and controlled clinical trials (CCT) to assess the effects of chemoprophylaxis and its effectiveness in preventing cholera in patient contacts.

## Materials and Methods

We conducted structured searches in PubMed (1966 to July 31, 2011), Cochrane Central Register of Controlled Trials (Central) (6; 2011), LILACS (1982 to July 31, 2011), Embase (1980 to July 31, 2011), and Scirus (July 31, 2011) (Annex 1). We searched the International Clinical Trials Registry Platform search portal of WHO (ICTRP) to identify past and ongoing trials using the key words “cholera” or “cholerae”. References of identified publications were screened and relevant websites were searched (document S1). We reached out to authors and relevant key stakeholders to identify unpublished studies and related additional data from manuscripts.

### Inclusion criteria and outcomes

We included randomized clinical trials (RCT) and controlled clinical trials (CCT) which assessed the effectiveness of antibiotics in preventing cholera among patients' contacts. We only considered RCTs/CCTs using chemoprophylaxis for contacts (children and/or adults) compared with no intervention, placebo, or other chemoprophylaxis regimens. Studies in which the intervention group included vaccines were excluded.

Clinical and bacteriological diagnoses of cholera (secondary cases) were the main outcomes considered in the review regardless of the microbiological method used to diagnose cholera. We included those adverse events reported in CCT/RCTs and did not search for additional adverse event studies or records. Due to variations in outcome reporting, we collected data which provided the number of subjects having at least one positive sample, the number of total subjects as well as the total number of positive samples, and the total number of samples obtained during follow-up. In addition, we collected data from trials that included or excluded contacts with positive samples on the first day of treatment. Findings are presented according to categories that were pre-specified by the trial.

### Study selection

Two independent reviewers assessed separately the titles, abstracts, and studies identified in the literature search (LR, EC). All trials matching the inclusion criteria were screened by two reviewers. Disagreements between reviewers were to be resolved by consensus or eventually majority by involving a third author; eventually the third author option was not required.

### Data extraction

At least two reviewers (LR, EC, TPK, PRP) independently extracted the relevant data using a predesigned data extraction form; disagreements between reviewers were resolved by referring to a third author. Key variables that were collected included identification data of the paper, treatment characteristics (type of intervention, dose, and duration), demographic variables (age, gender), number of participants, patients lost to follow up, proportion of patients with laboratory diagnoses of cholera (secondary cases), the methodology of the diagnosis, and all reported adverse events. Included studies were used to analyze the type and frequency of adverse effects, we only used included studies.

We performed an evaluation on the risk of bias for each trial following the Cochrane Collaboration tool for the assessment of these variables [12]. We also assessed inclusion and exclusion criteria; sample size calculation; and baseline comparability of age, gender, relevant clinical characteristics, and diagnoses. We registered data in the studies table (Table S1).

We obtained outcomes data from published articles and compared it using Review Manager Version 5.0 (RevMan 5). We contacted authors to obtain additional information about their studies.

### Statistical analysis

We used the RevMan 5 software from the Cochrane Collaboration to assist the statistical analysis. For dichotomous primary outcomes the results, expressed as relative risk (RR) and 95% confidence intervals (CI), were calculated using the Mantel–Haenszel random effects model. For the pooled analysis we calculated the I square (*I* ^2^) statistic that describes the percentage of total variation across studies attributed to heterogeneity [12]; low, moderate, and high levels of heterogeneity are roughly estimated as *I* ^2^ values of 25%, 50%, and 75%, respectively. Where necessary, multiple arms of a study were combined grouping the relevant interventions into a single group, and all relevant control interventions as a single control group [12]. The PRISMA check list is provided as suppoting information (Document S2).

## Results

### Characteristics of studies

**Figure 1:**
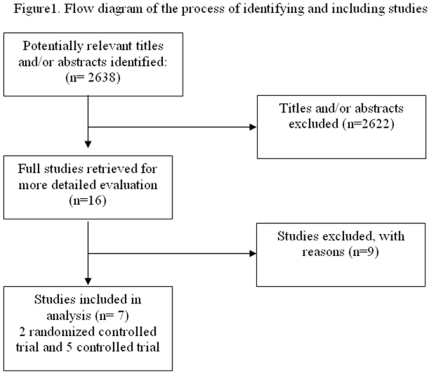
Flow diagram of study selection process.

We identified 2638 references of interest (Figure 1) through the literature search and deemed relevant 16 studies of chemoprophylaxis amongst contacts of individuals diagnosed with cholera. We included and analyzed 7 trials: two randomized controlled trials (RCTs) and five controlled trials (CCTs), with 4.154 participants altogether (2606 of them received chemoprophylaxis) (Table S1) [13]–[19]. Nine references did not comply with eligibility criteria and were excluded (Table S2) [10], [20]–[27].

Three CCTs were conducted in India [13], [15], [19]; the remaining four CCTs were in Bangladesh [16], Pakistan [18], Ivory Coast [17] and Peru [14]. One CCT allocated families, rather than individuals, to prophylaxis [18]. One of the seven trials was published in the 1960s [18], four in the 1970s [13], [15], [17], [19], one in the 1980s [16], and one in the 1990s [14]. The follow-up time (range 8 to 15 days), as well as the number of stool samples collected (range 0 to 15), varied among studies. A number of studies included contacts having positive bacteriological cholera on the first day, while others excluded such contacts. Outcomes were also reported in various formats across the studies; few trials reported clinical outcomes (Table S1).

### Assessment of risk of bias

Overall the quality of the reporting and design of the RCTs was poor (Table S3). Two studies were randomized controlled trials [14], [17] and five were controlled clinical trials [13], [15], [16], [18], [19]. We found one RCT with unclear risk of bias [14] and the remaining six trials were scored as having a high risk of bias mainly because of inadequate methods to generate the sequence of randomization (alternation), lack of allocation concealment, and/or incomplete outcome data. One trial [16] included a “no treatment” arm with most patients receiving vitamins instead of an inert placebo [13], [15], [17]–[19]. One study [17] reported data only for those contacts that developed cholera. The majority of trials did not provide a sample size framework and a scientific rationale for the sample size determination.

### Effects of Interventions

#### Overall chemoprophylaxis

The seven trials assessed the effects of different medications (tetracycline, doxycycline, ciprofloxacine, sulfadoxine) vs. vitamins (used as placebo) or no treatment. They also determined effectiveness based on different outcomes; data for each individual outcome were not available in all trials (Table S4). Only one RCT used an adequate placebo [14].

#### Clinical outcomes

**Figure 2:**
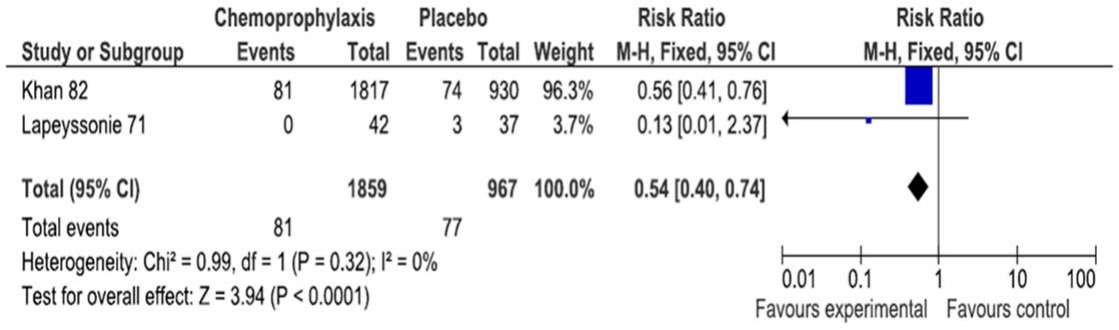
Prevention of hospitalization of contacts of cholera patients during the follow-up.

Few trials reported data on clinical outcomes, including adverse effects (Table S4). No reports of mortality were found in any trial. When we pooled two trials [16], [17], chemoprophylaxis (which included tetracycline and sulfadoxine) was significantly more effective in preventing hospitalization of contacts during the follow-up (2826 participants; RR 0.55 95% CI 0.41 to 0.75; I^2^ 0%; Figure 2). However, both RCTs did not provide sufficient information to determine if the authors reported all cause hospitalizations or only cholera related hospitalizations.

#### Bacteriological outcomes

**Figure 3:**
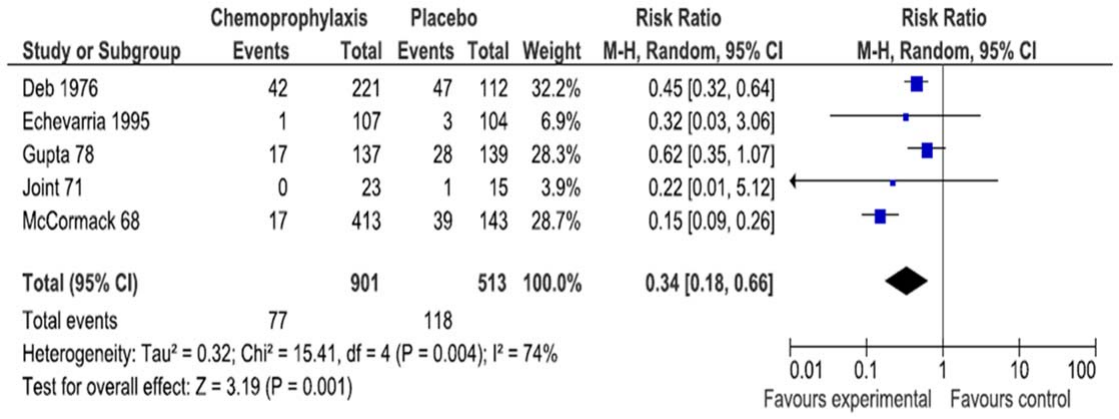
Proportion of patients with at least one positive cholera sample during the overall follow-up.

**Figure 4:**
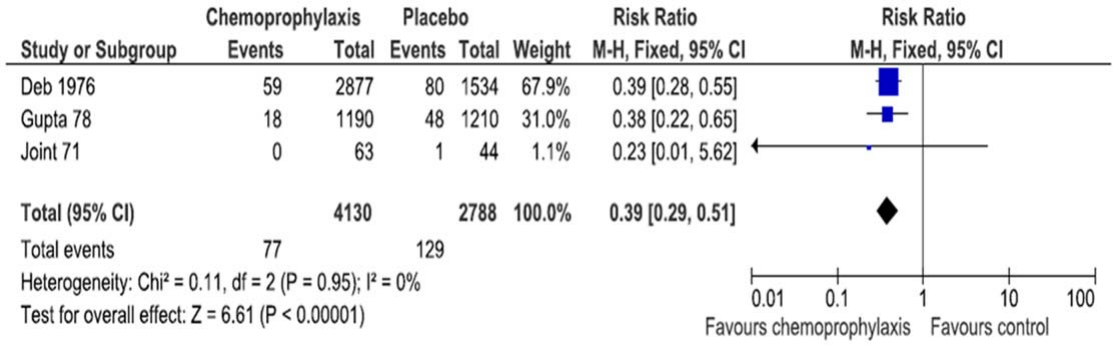
Proportion of total positive cholera samples/total number of samples during the overall follow-up.

Meta-analysis of five trials [13]–[15], [18], [19] found a significant reduction in the rate of at least one positive sample during the overall follow-up amongst contacts receiving chemoprophylaxis compared with the control group (1414 participants; RR 0.34 95% CI 0.18 to 0.66; Figure 3); there was significant heterogeneity among the 5 studies (I2 = 74%, P<0.01). Chemoprophylaxis was also found to reduce the total number of positive samples (one patient could have multiple positive samples on different days) (3 trials; 6918 samples; RR 0.39 95% CI 0.29 to 0.51;I^2^ 0%; Figure 4) [13], [15], [19]. When positive samples of contacts from day one during the reported follow-up period were included, we found that chemoprophylaxis reduced the rate of contacts presenting at least one positive sample (2 trials, 832 participants; RR 0.40 95% CI 0.14 to 1.11;I^2^ 82%) [18], [19] and the overall rate of positive samples (4 trials, 8.885 samples; RR 0.34 (0.20 to 0.59;I^2^ 64%) [13]–[15], [19]. Meta-analysis of trials that reported data on 4, 7, and 15 days of follow-up are shown in Table S4. When considering only trials assessing the effects of tetracycline and doxycycline, the combination of tetracycline and doxycycline prevented contacts from having positive samples, as compared with placebo, and reduced the proportion of at least one positive sample amongst contacts (Table S4).

#### Vibrio cholera susceptibility

One RCT [14] reported that *V. cholera* 01 strains isolated from index cases and household contacts had *in vitro* susceptibility to ciprofloxacin (MIC, 0.007 µg/mL). One CCT [13] concluded that all the strains of *V. cholera* isolated during the study were biotype El Tor, with no indications of emergence of resistant strains when sulfadoxine was used as a single dose.

#### Adverse events

Few trials reported adverse events [13], [14], [19] and none assessed systematically the specific adverse effects observed. Reports noted that adverse events were mild, but criteria and/or judgment with regards to categorization of the severity were not explicit. In combining the data of two trials [13], [14] that assessed utcomes after two weeks of treatment, we found no significant difference in the incidence of diarrhea; one CCT [19] reported that 7.3% of contacts that received doxycycline complained of nausea, and 2.9% suffered from mild vomiting.

## Discussion

### Main Findings

This systematic review suggests that hospitalization of contacts of cholera patients can be reduced by 26% to 60% with the prophylactic administration of antimicrobial agents (the hospitalization rate was 4.4% versus 8.0% in the control group). The studies available for analysis are a highly heterogeneous group that assessed different interventions and different outcomes with different methods and at different follow-up times, with a variety of sampling methods and outcome reporting. Most of the studies focused on microbiological outcomes, documenting the excretion of V. cholera after chemoprophylaxis [13]–[15], [18], [19], but there is limited information about impact. Household contacts were followed for short periods of time. It is uncertain whether those who received chemoprophylaxis ultimately had the same rates of cholera and hospitalization compared to those in the control group. Other limitations of this review mainly concern the methodological quality and poor reporting of most of the included studies. Because most studies were conducted during the 1960's and 1970's, it is likely that the epidemiological conditions (e.g.resistance, dissemination patterns) have changed. The external and internal validity of included studies is likely compromised by factors that increase bias and imprecision, with a failure to address clinically important outcomes and adequate assessment of the balance between benefits and harms for participating individuals or populations. Most clinical trials included in the review were performed at a time when antibiotic resistance to cholera was rare, although a number of studies worldwide suggest an increase in resistance of V. cholera to several antibiotics worldwide [28]–[41]. Some of those studies strongly suggest that mass chemoprophylaxis, and nonselective chemoprophylaxis of adult family members, can contribute to the emergence of resistance [38]–[40]. In addition, attempts to provide chemoprophylaxis to household contacts require resources and time, and may have associated risks and result in unexpected harms. It is difficult with the available data to draw conclusions that could be adequately used to inform policy and practice.

The containment of cholera needs adequate scientific basis. This review highlights knowledge gaps and provides recommendations for future studies to address the impact of chemoprophylaxis in crowded or enclosed settings (e.g. prisons, camps, nurseries, orphanages, nursing homes, and ships). Future studies should evaluate the effectiveness of relevant existing and new single dose therapeutic options (e.g. azythromycin). The effectiveness of chemoprophylaxis in reducing hospitalization rates should be confirmed in well designed and conducted RCTs of adequate sample size. Longer follow-up periods than in the past studies are needed to determine the effectiveness of antibiotics. It seems likely that only large cluster randomized trials, using an appropriate placebo can provide the data required to make informed decisions regarding the use of prophylactic antibiotics in cholera outbreaks. RCTs should also conform to the guidelines from the Consolidated Standards of Reporting Trials (CONSORT) statement [42].

## Supporting Information

Table S1 **Characteristics of studies included in the review.**
(DOC)

Table S2 **Studies excluded from the review.**
(DOC)

Table S3 **Assessment of bias risk of randomized & controlled clinical trials.**
(DOC)

Table S4 **Main outcomes of clinical trials.**
(DOC)

Document S1 **Search strategies.**
(DOC)

Document S2 **Prisma check list.**
(DOC)
