# Supplementary material for: Chemoprophylaxis in Contacts of Patients with Cholera: Systematic Review and Meta-Analysis
Source: PLoS One. 2011 Nov 15;6(11):e27060. doi: 10.1371/journal.pone.0027060 (PMC3216950; doi:10.1371/journal.pone.0027060)
Supplement: Table S1 — Characteristics of studies included in the review. (DOC) [file pone.0027060.s001.doc]

**Table S1. Characteristics of studies included in the review**

| **Study** | **Methods** | **Participants** | **Interventions** | **Outcomes** |
| --- | --- | --- | --- | --- |
| Deb 1976 (13) | Controlled trial. The study population was divided into groups who received sulfadoxine, tetracycline, or multivitamins (placebo). Household contacts enrolled in the study were given sequential study numbers. | India  The study population consisted of household contacts of index cases with diarrhea caused by V. cholera who attended at Infectious Diseases Hospital of Calcutta. They were visited within 24 hours of the onset of symptoms of the index cases and samples of stools/rectal swabs were collected from all family members.  Inclusion criteria: all household contacts (above 1 year) for which the index cases were bacteriologically confirmed.  Exclusion criteria: Infants below 11/2 years and contacts of the index cases that were subsequently reported to be bacteriologically negative. | Sulfadoxine  1-1/2 to 4 years: 0.5 g once only.  5-14 years: 1.0 g once only.  >14: 2.0 g once only  Tetracycline  1-1/2 to 12 years: 0.25 g every 12 h for 3 days  > 12 years: 0.5 g every 12 h for 3 days  Multivitamins were administered to the placebo group. | Effectiveness: carriers detected during the 15-day follow-up  (effectiveness between males and females and by age)  Adverse reactions |
| Echevarria 1995 (14) | Randomized double-blinded study. Randomization using a random table with fixed blocks containing 50 numbers. Household contacts enrolled in the study were given sequential study numbers. The team members and the household contacts were blinded. | Peru.  The study population consisted of household contacts of index cases with diarrhea caused by V. cholera 0 1 El Tor who attended the emergency ward at the National Hospital Cayetano Heredia in Lima, Peru. Inclusion criteria: adult household contacts (18- 65 years) having baseline stool cultures negative for V. cholera 0 1 El Tor. Exclusion criteria: a history of watery diarrhea within the previous 2 years; allergy to quinolones;  Pregnancy or breast-feeding; receipt of antibiotics active against Vibrio within 7 days before enrollment; and the need for drugs other than those provided in the study. | Single 250-mg tablet of ciprofloxacin  or placebo | Clinical prevention: absence of diarrhea during the 14 days of the study period. Diarrhea was defined as three or more loose stools purged during 24 hours  Microbiological Prevention: negative cultures for V. cholera  01 from the samples taken on the 3rd and 7th days of follow-up. |
| Joint ICMR-GWB-WHO 1971 (15) | Controlled double blind trial | India.  The study population consisted of households in  Calcutta that had hospitalized cholera cases during the epidemic period of 1969. Stool samples were collected from each member of the household on the first day within 24-48 hours of onset of the disease of the index case; all household contacts were included. Convalescent cholera patients were also included in the study if they were discharged during the study period, and were assigned to the same group as their household. | Tetracycline or a placebo (multivitamins). Tetracycline administered orally in syrup or capsules, depending on the age every 10-12 hours for 3 consecutive days. >13 years 500 mg every 12 hours. 4-12 years 250 mg every 12 hours. <3 125 mg every 12 hours. | Microbiological Prevention: Stool samples were taken from contact; rectal swabs were collected from children under 5 years of age and from adults if stool samples were not available. All the household contacts and discharged cholera patients were sampled every day for 10 days |
| Khan 1982  (16) | Controlled trial. Selected alternatively. | Bangladesh.  Contacts of hospitalized confirmed cholera cases.  Exclusion criteria: not reported | Tetracycline 250 mg  Twice or nothing | Hospitalization for severe diarrhea and diarrhea. Families were visited 10 to 12 days after medication. |
| Lapeyssonnie 1971  (17) | Randomized double-blinded study and using a random table. | Côte d'Ivoire  Almost all population (506/600 hab) of Lake Ebrie of Abidjan (Côte d'Ivoire) | Sulphometoxine (1 dose)  1,5-4 ys= 0,5 gr  5-9 ys=1 gr  10-14 ys= 1,5 gr  >15 ys= 2 gr  Or placebo (Pyridoxine) | Carriers/diarrhoea  Hospitalization. A contacts were sampled every day for 8 days |
| McCormack 1968  (18) | Controlled trial. Families were selected alternatively to one of 4 groups. | Pakistan  Families of patients admitted to the Pakistan-SEATO Cholera Research Laboratory (PSCRL) hospital in Dacca from December 1966 to February 1967.  Inclusion: families with more than 3 members in which the index patient was the first in the family to have cholera. Convenience of geographical location as well as willingness to co-operate was considered.  Exclusion: Families in which the index patient was subsequently found not to have cholera were dropped from the study. | Total daily dose of tetracycline in the treated groups was 1.0 g for those 10 years of age or older and 500 mg for those under 10 years of age.  Placebo | Microbiological  Culture positive by day and drug dosage. The team visited each family daily for a total of 10 days. |
| Sen Gupta 1978 (19) | Controlled trial. The study population was divided into groups who received either intervention or placebo. Household contacts enrolled in the study were given sequential study numbers. | India (Calcutta)  Inclusion criteria: Stool samples or rectal swabs of family contacts of cholera index cases (bacteriologically established)  Exclusion criteria: Infants under 3 months of  age were excluded from the study. | Doxycycline.  300 mg and 6 mg/kg/dose (<15 y) or Placebo (Multivitamins) | Effectiveness after administration in reducing the load of infection among family contacts minor reactions. All 276 contacts were examined bacteriologically daily for 10 days for the presence of V. cholera in their stools. |
