# Supplementary material for: Chemoprophylaxis in Contacts of Patients with Cholera: Systematic Review and Meta-Analysis
Source: PLoS One. 2011 Nov 15;6(11):e27060. doi: 10.1371/journal.pone.0027060 (PMC3216950; doi:10.1371/journal.pone.0027060)
Supplement: Table S2 — Studies excluded from the review. (DOC) [file pone.0027060.s002.doc]

**Table S2. Studies excluded from the review**

| **Study** | **Reason for exclusion** |
| --- | --- |
| Gharagozloo 1970 | Trial on treatment rather than on chemoprevention |
| Pierce 1968 | Trial on treatment rather than on chemoprevention |
| Usubütün 1997 | Trial on treatment rather than on chemoprevention |
| Islam 1987 | Trial on treatment rather than on chemoprevention |
| Sack 1978 | Trial on treatment rather than on chemoprevention |
| Guévart 2004 | Non controlled study |
| Guévart 2006 | Non controlled study |
| Guévart 2007 | Non controlled study |
| Gangarosa 1966 | Trial on treatment rather than on chemoprevention |
