# Supplementary material for: Chemoprophylaxis in Contacts of Patients with Cholera: Systematic Review and Meta-Analysis
Source: PLoS One. 2011 Nov 15;6(11):e27060. doi: 10.1371/journal.pone.0027060 (PMC3216950; doi:10.1371/journal.pone.0027060)
Supplement: Table S3 — Assessment of bias risk of randomized & controlled clinical trials. (DOC) [file pone.0027060.s003.doc]

**Table S3. Assessment of bias risk of randomized & controlled clinical trials^a^**

| **Study** | **Sequence generation** | **Allocation concealment** | **Blinding of participants, personnel, and outcome assessors** | **Incomplete outcome data and withdrawals** | **Free of selective reporting?** | **Other sources of bias and commentaries** | **Overall assessment**  **High/ moderate/ low risk of bias** |
| --- | --- | --- | --- | --- | --- | --- | --- |
| Deb 1976 | No  Assigned by strict alternation | No | Unclear  “Persons in the placebo group also received 6 doses of multivitamins to match the dosage schedule of tetracycline”. | No | No  Clinical outcomes not included | Unclear  No conflict of interest was reported. | High |
| Echevarria 1995  (Main author was contacted and provided further information on methods) | Yes  Random table with fixed blocks containing 50 numbers | Yes  Household contacts enrolled in the study were given sequential study numbers. It was not possible for either the team members or the household contacts to identify the study drugs. Information provided by the author: codes were assigned centrally. | Yes  The study drug was provided in a sachet labeled only with the study number. Information provided by the author: Personnel and patients were blind; the medication and placebo had similar appearance. Codes were open once the trial was terminated. | Unclear  250 were randomized; of these, 213 fulfilled all the inclusion criteria  (20 from the placebo group and 17 from the ciprofloxacin group were excluded). Two additional household contacts were excluded from the efficacy analysis (one voluntarily withdrew and one was excluded because she received other treatment). Cholera samples were taken only the 3rd and 7th days of follow-up. | Unclear | Unclear  The study was partially financed by Bayer's Latin  American Affairs, Bayer-México. Conflict of interests not declared.  Informed Consent, protocol approved by Ethical IRB of Universidad Peruana Cayetano Heredia. | Unclear |
| Joint ICMR-GWB-WHO 1971 | Unclear  Probably not done | Unclear  Probably not done | Unclear  “In order to avoid  bias, the study was designed as a doubled-blind  Trial” | No  No clinical outcomes were collected. No adverse events reported. No baseline characteristics reported. | No  Clinical outcomes not included | Unclear  Conflict of interests not declared. | High |
| Khan 1982 | No  Assigned by strict alternation | No | No | No  Half of control group was not re-visit at 10-12 days  Contact participants were not tested for V. Cholera | No | Unclear  No conflict of interest was reported. | High |
| Lapeyssonnie 1971  (Preliminary report. Unclear authorship | Yes  Random Table | Unclear  Probably not done | Unclear  “Double blind”. It is unclear who was blind. No information relating to whether the intended blinding was effective. | No  Only data from contacts that were positive to V. Cholera the first day was included; data on 30% of them was not reported. No adverse events. | No | Unclear  No conflict of interest was reported. | High |
| McCormack 1968 | No  Assigned by strict alternation to one of 4 groups | No | No | Unclear  Data from 556 data of 655 contacts was reported. No information on difference of losses according to groups. More families in the groups given placebo or one single dose of tetracycline hydrochloride had at least I member (other than the index patient) infected | No  Clinical outcomes not included | Unclear  Differential treatment because the RAMADAN dosage schedule was modified.  No conflict of interest was reported. | High |
| Sen Gupta 1978 | No | No | Unclear  Although the term “placebo” was used, there is no description that the trial was “blind”. | Unclear.  Of the expected total of 2760 samples from 276 contacts, 2672 (96.8 %) were collected. | No  Clinical outcomes not included | Unclear.  No conflict of interest was reported. The Vibramycin brand of doxycycline used in the present trial was supplied  by the Pfizer Chemical Co. | High |

^a^ According to the Cochrane Collaboration Handbook:

Sequence generation: Was the allocation sequence adequately generated?

Allocation concealment: Was allocation adequately concealed?

Blinding of participants, personnel, and outcome assessors: Was knowledge of the allocated intervention adequately prevented during the study?

Incomplete outcome data and withdrawals: Were intention-to-treat analyses performed? Had participants withdrawn from the study?

Free of selective reporting?

Other sources of bias and commentaries: Was sample size calculated? Were inclusion and exclusion criteria and baseline characteristics defined? Were conflicts of interests reported?

^b^ Yes = low risk of bias.

^c^ No = high risk of bias.
