# Supplementary material for: Chemoprophylaxis in Contacts of Patients with Cholera: Systematic Review and Meta-Analysis
Source: PLoS One. 2011 Nov 15;6(11):e27060. doi: 10.1371/journal.pone.0027060 (PMC3216950; doi:10.1371/journal.pone.0027060)
Supplement: Table S4 — Main outcomes of clinical trials. (DOC) [file pone.0027060.s004.doc]

**Table S4. Main outcomes of clinical trials**

| **Study** | **Main Outcome** | **Intervention (n)** | **Comparator (n)** | **Estimate (95% CI)a** |
| --- | --- | --- | --- | --- |
| Khan 1982 (16)  Lapeyssonie 1971  (17) | Hospitalization during the follow (including contacts having positive cholera samples from day 1; including any follow-up period) | Chemoprophylaxis¶  1859 | Placebo  967 | RR 0.54 (0.40 to 0.74; I2 0%) |
| Deb 1976 (13)  Echeverria 1995 (14) | Diarrhea during 15 days | Chemoprophylaxis  317 | Placebo  216 | RR 0.64 (0.39 to 1.06; I2 0%) |
| Deb 1976 (13)  Joint 1971 (15)  Sen Gupta 1978 (19) | Total number of positive samples / total number of samples (one patient could have multiple positive samples in different days ^c^; including any follow-up period) | Chemoprophylaxis¶  4130 | Placebo  2788 | RR 0.39 (0.29 to 0.51; I2 0%) |
| Deb 1976 (13)  Echeverria 1995 (14)  Joint 1971 (15)  McCormack 1968 (18)  Sen Gupta 1978 (19) | Total number of patients with at least one positive sample (including any follow-up period). | Chemoprophylaxis¶  901 | Placebo  513 | RR 0.35 (0.18 to 0.66; I2 74%) § |
| Deb 1976 (13)  Echeverria 1995 (14)  Joint 1971 (15)  Sen Gupta 1978 (19) | Total number of positive samples / total number of samples during the follow-up (one patient could have multiple positive samples in different days ^c^; including contacts having positive cholera samples from day 1; including any follow-up period) | Chemoprophylaxis¶  5172 | Placebo  3713 | RR 0.34 (0.20 to 0.59; I2 64%) § |
| McCormack 1968 (18)  Sen Gupta 1978 (19) | Total number of patients with at least one positive sample (including contacts having positive cholera samples from day 1; including any follow-up period). | Chemoprophylaxis  550 | Placebo  282 | RR 0.40 (0.14 to 1.11; I2 82%) § |
| Deb 1976 (13)  Lapeyssonie 1971 (17)  McCormack 1968 (18) | Total number of patients with at least one positive sample during 2 weeks of follow-up | Chemoprophylaxis¶  730 | Placebo  359 | RR 0.28 (0.10 to 0.75;I2 84%) § |
| Deb 1976 (13)  Lapeyssonie 1971 (17)  Sen Gupta 1978 (19) | Total number of positive samples / total number of samples during 7 days of follow-up (one patient could have multiple positive samples in different days ^c^; including contacts having positive cholera samples from day 1; including any follow-up period) | Chemoprophylaxis¶  2671 | Placebo  1935 | RR 0.33 (0.26 to 0.42; I2 0%) |
| Joint 1971 (15)  McCormack 1968 (18)  Sen Gupta 1978 (19) | Total number of patients with at least one positive sample during 4 days of follow-up. | Chemoprophylaxis¶  841 | Placebo  564 | RR 0.12 (0.05 to 0.25; I2 0%) |
| Deb 1976 (13)  Joint 1971 (15)  Sen Gupta 1978 (19) | Total number of positive samples /total number of samples during the follow-up (one patient could have multiple positive samples in different days ^c^; including any follow-up period) | Tetracycline/doxycycline¶  2737 | Placebo  2788 | RR 0.38 (0.28 to 0.53; I2 0%) |
| Deb 1976 (13)  Joint 1971 (15)  McCormack 1968 (18)  Sen Gupta 1978 (19) | Total number of patients with at least one positive sample (including any follow-up period; tetracycline 3 to 5 days of treatment). | Tetracycline/doxycycline¶  659 | Placebo  409 | RR 0.26 (0.09 to 0.77; I2 84%) § |

^a^ CI = confidence interval.

^b^ RR = relative risk.

^c^ Total number of positive samples / total number of samples during follow up.

¶ favoring this intervention

§ For heterogeneity > 49%, the random effect model was used.
