# Supplementary material for: Chemoprophylaxis in Contacts of Patients with Cholera: Systematic Review and Meta-Analysis
Source: PLoS One. 2011 Nov 15;6(11):e27060. doi: 10.1371/journal.pone.0027060 (PMC3216950; doi:10.1371/journal.pone.0027060)
Supplement: Document S1 — Search strategies. (DOC) [file pone.0027060.s005.doc]

**Search strategies**

**PubMed (July 31, 2011)**

(Cholera [mh] OR Cholera* [tw]) AND (Chemoprevention [mh] OR chemoprophyla* [tw] OR prophyla* [tw] OR Household* OR Contact* [tw] OR Famil* [tw] OR relative* [tw]) AND (randomized controlled trial[pt] OR controlled clinical trial[pt] OR randomized[tiab] OR placebo[tiab] OR drug therapy[sh] OR randomly[tiab] OR trial[tiab] OR groups[tiab] OR "treatment studies"[tiab] OR "therapy studies"[tiab]) NOT (animals[mh] NOT (humans[mh] AND animals[mh]))

**Lilacs (July 31, 2011)**

Tw estud$ OR Tw clin$ OR AB grupo$ OR CT COMPARATIVE STUDY OR Tw placebo$ OR Tw random$ OR Ti compara$ OR Ti tratamiento OR Tw control$ OR MH /dt [Words] and MH CHOLERA OR Tw CHOLERA$ [Words]

**Embase (1980 to July 31, 2011)**

#32 #1 AND #29 AND #30 AND #31

#31 #8 OR #9 OR #10 OR #11 OR #12 OR #13 OR #14

#30 #2 OR #3 OR #4 OR #5 OR #6

#29 #25 NOT #28

#28 #26 NOT #27

#27 'human'/syn AND [embase]/lim

#26 'animals'/exp AND [embase]/lim

#25 #17 OR #18 OR #19 OR #20 OR #21 OR #22 OR #23 OR #24

#24 groups:ab AND [embase]/lim

#23 trial:ab AND [embase]/lim

#22 randomly:ab AND [embase]/lim

#21 'drug therapy'/syn AND [embase]/lim

#20 placebo:ab AND [embase]/lim

#19 'controlled clinical trial'/exp AND [embase]/lim

#18 'randomized controlled trial'/exp AND [embase]/lim

#17 randomized:ab AND [embase]/lim

#14 'relative'/exp AND [embase]/lim

#13 relative* AND [embase]/lim

#12 'family'/exp AND [embase]/lim

#11 famil* AND [embase]/lim

#10 contact* AND [embase]/lim

#9 household* AND [embase]/lim

#8 'household'/exp AND [embase]/lim

#6 'prophylaxis'/exp AND [embase]/lim

#5 prophyla* AND [embase]/lim

#4 'chemoprophylaxis'/exp AND [embase]/lim

#3 chemoprophyla* AND [embase]/lim

#2 'chemoprevention'/exp AND [embase]/lim

#1 'cholera'/exp AND [embase]/lim

**Scirus (Limits: medicine; July 31, 2011)**

(title:cholera OR cholerae) AND (households OR households OR contacts OR family OR contact) AND (randomized OR randomised OR random OR trial)

**Cochrane Central Register of Controlled Trials (6; 2011);**

Cholera OR Cholerae (Mesh and text word)

**International Clinical Trial Platform Registry (July 31, 2011)**

Cholera OR Cholerae

**Relevant websites**

WHO AFRO Library; WHO | Index Medicus for the Eastern Mediterranean Region; IMSEAR; Index Medicus for South-East Asia Region; WHO Western Pacific Region; and TropIKA.
